# Editorial: The Role of Metabolism in MSC-Mediated Immunomodulation

**DOI:** 10.3389/fimmu.2021.751865

**Published:** 2021-08-26

**Authors:** Yves-Marie Pers, Christian Jorgensen, Maroun Khoury

**Affiliations:** ^1^IRMB, University of Montpellier, INSERM, CHU Montpellier, Montpellier, France; ^2^Clinical Immunology and Osteoarticular Diseases Therapeutic Unit, Department of Rheumatology, Lapeyronie University Hospital, Montpellier, France; ^3^Laboratory of Nano-Regenerative Medicine, Faculty of Medicine, Universidad de los Andes, Santiago, Chile; ^4^Cells for Cells and REGENERO, Chilean Consortium for Regenerative Medicine, Santiago, Chile

**Keywords:** MSC, metabolism, immunomodulation, extracellular vesicles (EV), miR (microRNA), macrophages, glycolytic activity, mitochondria transfer

Multipotent mesenchymal stem/stromal cells (MSCs) are progenitor cells and exert several functions including support of hematopoiesis, regeneration, resistance to fibrosis, apoptosis or hypertrophy. MSCs have also immunomodulatory and immunosuppressive properties that may explained various positive results in cell therapy for inflammatory diseases (1). MSC can affect both the innate and the adaptive immunity (2). This immunosuppressive effect is mainly due to the secretion of soluble factors by MSCs and by direct contact with immune cells (3). Recent advances have established that plasticity of immune functions occurs in distinct metabolic stress features. Evidence has accumulated to indicate that specific metabolic signatures dictate appropriate immune functions in both innate and adaptive immunity (4). Recently, it has been shown that manipulating metabolic pathways of cancer cells, T-cells or immune suppressor cells can enhance anti-cancer immunity and suppress tumor growth (5, 6).

Our Research Topic aimed to cover promising, recent, and novel research trends characterizing MSC metabolism, deciphering MSC immunomodulatory properties related to energy metabolism and guiding future perspectives for therapeutic applications with MSC. The different contributors achieved these objectives by providing new insights with original articles and high quality reviews. MSCs can be considered as major actors of the immune system orchestra, and play a strategic role in the repair mechanisms *via* a pro-inflammatory and anti-inflammatory response, in particular by driving the macrophages polarization with an activation of glycolytic pathways (Luque-Campos et al.; Planat-Benard et al.). Cellular metabolic fluxes and metabolite detection dictate the immune response developed by macrophages, dendritic cells, neutrophils and lymphocytes. The metabolism of MSCs can be modulated to improve their functional properties. MSCs respond to damaged or inflamed tissue through the transfer of Mitochondria (MT) to injured and immune cells, conveying a type of signaling that contributes to the restoration of cell homeostasis and immune function. MT offers interesting therapeutic perspectives *via ex vivo* treatments (AMPK activation, allogeneic transfer of BMSCs from healthy donors) even if several challenges remain (Jorgensen and Khoury; Loussouarn et al.). Moreover, recent studies have shown that miRNAs are able to translocate into the mitochondrial compartment and modulate mitochondrial activities. In particular, the miR-155/miR-221 axis acts as a new player in the immunoregulatory function of human BMSCs (Pers et al.). MSC-derived EVs (containing and releasing miRNAs) may become alternative treatment in regenerative medicine as they can promote mitochondrial function (Loussouarn et al.). In addition, the different authors outlined the main biological processes with therapeutic prospects: cancer, wound healing, regeneration and inflammation/autoimmunity. Graft versus host disease (GVHD) was the first autoimmune disease model demonstrating a benefit using BMSC (7) and leading to a large development of clinical trials in several indications (inflammatory bowel diseases, lupus, rheumatoid arthritis, multiple sclerosis). Indeed, MSC-based cell products have now been approved for the treatment of acute GVHD in pediatric patients in Japan, Canada and New Zealand (Burnham et al.), and for peri-anal Crohn’s disease (8). Burnham et al. highlighted the key roles of IFN-γ, IDO, PGE2, HIF1α, HO-1, as well as energy-generating metabolic pathways in GVHD. In addition to soluble factors, cell-to-cell contact is involved in the inhibition of T cell proliferation by MSCs and a new mechanism through the ICAM-1/CD43 interaction has been described in a GHVD model as well (Zheng et al.). The therapeutic use of MSCs raises many questions. Indeed, the use of allogeneic MSCs presents several advantages compared to autologous MSCs (lower production costs, simplification of the study methodology). However, the induction of humoral and/or cellular alloimmunity by allogeneic MSCs could limit their therapeutic efficacy and could cause adverse effects, if injections are repeated (9, 10). In addition, we still need to explore the best culture conditions, choose the best MSC source (ASC, BM-CSM, umbilical cord), since metabolism changes may happen according to the cell source (Jeske et al.).

To conclude, cellular metabolism and immunity are intertwined and must be considered together. The influence of these metabolic changes on MSC functional properties starts to emerge. Therefore, a clearer understanding of how MSC metabolism impacts immunomodulatory function will have a large interest, especially for futures MSC-based therapies, including EVs and mitochondria. Indeed, targeting cellular metabolism has and will become an attractive target for the development of new therapeutics. 

## Author Contributions

Y-MP, CJ, and MK contributed to conception and design of the study. Y-MP wrote the first draft of the manuscript. All authors contributed to the article and approved the submitted version.

## Conflict of Interest

The authors declare that the research was conducted in the absence of any commercial or financial relationships that could be construed as a potential conflict of interest.

## Publisher’s Note

All claims expressed in this article are solely those of the authors and do not necessarily represent those of their affiliated organizations, or those of the publisher, the editors and the reviewers. Any product that may be evaluated in this article, or claim that may be made by its manufacturer, is not guaranteed or endorsed by the publisher.
